# Combined action observation and imagery facilitates corticospinal excitability

**DOI:** 10.3389/fnhum.2014.00951

**Published:** 2014-11-27

**Authors:** David J. Wright, Jacqueline Williams, Paul S. Holmes

**Affiliations:** ^1^Institute for Performance Research, Manchester Metropolitan UniversityCrewe, UK; ^2^Institute of Sport, Exercise and Active Living and College of Sport and Exercise Science, Victoria UniversityMelbourne, VIC, Australia

**Keywords:** action observation, movement imagery, transcranial magnetic stimulation, motor evoked potentials, stroke rehabilitation

## Abstract

Observation and imagery of movement both activate similar brain regions to those involved in movement execution. As such, both are recommended as techniques for aiding the recovery of motor function following stroke. Traditionally, action observation and movement imagery (MI) have been considered as independent intervention techniques. Researchers have however begun to consider the possibility of combining the two techniques into a single intervention strategy. This study investigated the effect of combined action observation and MI on corticospinal excitability, in comparison to either observation or imagery alone. Single-pulse transcranial magnetic stimulation (TMS) was delivered to the hand representation of the left motor cortex during combined action observation and MI, passive observation (PO), or MI of right index finger abduction-adduction movements or control conditions. Motor evoked potentials (MEPs) were recorded from the first dorsal interosseous (FDI) and abductor digiti minimi (ADM) muscles of the right hand. The combined action observation and MI condition produced MEPs of larger amplitude than were obtained during PO and control conditions. This effect was only present in the FDI muscle, indicating the facilitation of corticospinal excitability during the combined condition was specific to the muscles involved in the observed/imagined task. These findings have implications for stroke rehabilitation, where combined action observation and MI interventions may prove to be more effective than observation or imagery alone.

## Introduction

Research using neuroimaging techniques (e.g., Grèzes and Decety, 2001; Filimon et al., 2007; Munzert et al., 2008) has indicated that several cortical areas shown to be active during movement execution are also active during the action observation and imagery of movement. These areas include the dorsal pre-motor cortex, primary motor cortex, supplementary motor area, superior parietal lobe, intraparietal sulcus, and cerebellum. Therefore, when physical movement is not possible, as in the case of stroke or other brain injury, action observation and imagery may provide useful techniques for maintaining activity in motor regions of the brain, and so assist in the recovery of motor functioning (Sharma et al., 2006; de Vries and Mulder, 2007; Ertelt et al., 2007; Holmes and Ewan, 2007; Mulder, 2007). As such, considerable research attention has been devoted to understanding the effects of action observation and imagery on the human motor system and establishing techniques for best utilizing action observation and imagery in rehabilitation settings.

One method that has been used to investigate the effects of action observation and imagery independently on the human motor system is transcranial magnetic stimulation (TMS). When TMS is applied to the primary motor cortex, motor evoked potentials (MEPs) are produced in the corresponding muscles; the amplitude of which provides a marker of corticospinal excitability at the time of simulation (Rothwell, 1997; Petersen et al., 2003; Naish et al., 2014). Research into action observation indicates that single-pulse TMS delivered to participants’ motor cortex during observation of human movements produces MEPs of larger amplitude than those obtained under control conditions (e.g., Fadiga et al., 1995; Strafella and Paus, 2000; Patuzzo et al., 2003; Borroni et al., 2005; Aglioti et al., 2008; Loporto et al., 2012). This indicates that passive observation (PO) of hand and arm movements can facilitate corticospinal excitability. A similar effect also occurs during imagery of human movements, where the amplitude of MEPs obtained during imagery are larger than those obtained under control conditions (e.g., Kasai et al., 1997; Fadiga et al., 1999; Hashimoto and Rothwell, 1999; Rossini et al., 1999; Facchini et al., 2002). Stinear et al. (2006), however, have reported that this effect is only present when participants engage in kinesthetic imagery, but not visual imagery.

As both action observation and imagery have been shown to facilitate corticospinal excitability, albeit through partially different neural mechanisms, several researchers have compared the facilitation effects of action observation and imagery in an attempt to establish which may be the more effective technique. For example, Clark et al. (2004) used TMS to stimulate the motor cortex representation for the right hand muscles during observation, imagery, and physical imitation of simple hand movements. In comparison to a resting control condition, both action observation and imagery produced a corticospinal facilitation effect, but there was no difference in the extent of the facilitation between the two experimental conditions. This effect has since been replicated consistently in the literature (e.g., Léonard and Tremblay, 2007; Roosink and Zijdewind, 2010; Williams et al., 2012), indicating that action observation and imagery facilitate corticospinal excitability to a similar extent.

Action observation and imagery have, therefore, traditionally been viewed as separate intervention techniques. Researchers have either studied the effects of action observation or imagery in isolation, or compared the effects of the two techniques against each other. More recently, it has been proposed that action observation and imagery should be viewed as complementary, rather than competing, interventions (Holmes and Calmels, 2008). Indeed, Vogt et al. (2013) have suggested that it is possible for humans to observe a movement whilst concurrently imagining that they are performing that same movement; a process they term “congruent action observation-motor imagery”. Given that both action observation and imagery activate the motor system when performed in isolation, it is logical to assume that combining the two techniques may activate the motor system to a greater extent. Recent fMRI and EEG research would support this assertion (e.g., Macuga and Frey, 2012; Nedelko et al., 2012; Berends et al., 2013; Villiger et al., 2013, for a review see Vogt et al., 2013). Collectively, this body of research has revealed that, compared to PO, concurrent action observation and imagery of a variety of congruent movement tasks produces stronger activation in several movement-related brain regions.

Single-pulse TMS has also been used to explore the effects of combined action observation and imagery on corticospinal excitability. For example, Sakamoto et al. (2009) stimulated the left motor cortex representation for the biceps brachii muscle whilst participants: (i) observed passively a bicep curl action; (ii) imagined performing a bicep curl action; or (iii) observed a bicep curl action whilst simultaneously imagining that they were performing that same action. The amplitude of MEP responses in these three conditions were compared to those obtained from a control condition, involving passive observation of a fixation cross. Both imagery alone and the combined action observation and imagery conditions produced larger amplitude MEPs than the control condition, in contrast to the PO condition. Importantly, the authors also reported that the combined action observation and imagery condition produced larger amplitude MEPs than either action observation or imagery conditions alone. Similar findings have also been reported by Ohno et al. (2011) and Tsukazaki et al. (2012) for combined observation and imagery of chopstick use and three-ball juggling in novices, respectively. Based on these findings, the authors suggested that combining action observation and imagery into a single intervention strategy may be more effective for aiding recovery of motor function in patients than either action observation or imagery alone. This argument is supported by the recent behavioral evidence provided by Eaves et al. (2014), which indicates that engaging in combined observation and imagery can facilitate subsequent motor execution.

Although all three combined action observation and imagery experiments that have been published to date using TMS have demonstrated that combined action observation and imagery produces larger amplitude MEPs than either action observation or imagery alone (e.g., Sakamoto et al., 2009; Ohno et al., 2011; Tsukazaki et al., 2012), the experiments were limited by a number of methodological factors. First, these experiments all used observation of a fixation cross or a blank screen as the control condition against which to compare MEP amplitudes obtained in the action observation and imagery conditions. Use of such a control condition is problematic in that it makes the interpretation of the corticospinal facilitation effect difficult (Loporto et al., 2011). Loporto et al. (2011) argued that by using a fixation cross or blank screen as the only control condition in TMS action observation and imagery experiments, researchers are unable to attribute accurately any facilitation effect to the specific observation and/or imagery task. For example, any facilitation effect found for action observation in comparison to a fixation cross or blank screen control, may be due to the presence of movement in the experimental condition rather than the specific observation of task-related human movement. Equally, facilitation effects obtained during imagery, in comparison to a fixation cross or blank screen control, may be due to participants engaging in any form of cognitive activity, rather than specific imagery of human movement. Taken together, it is important to conduct similar experiments for combined action observation and imagery whilst employing more rigorous control conditions, in order to ascribe accurately this effect to the experimental manipulation.

Further, in the reported combined action observation and imagery TMS studies (i.e., Sakamoto et al., 2009; Ohno et al., 2011; Tsukazaki et al., 2012) the ordering of trials was randomized by experimental condition across the experiment. Although such a randomization procedure is common in typical TMS action observation and imagery research, we argue that to do so in a combined action observation and imagery experiment is problematic. The video stimulus provided to participants is, typically, identical in the PO and combined action observation and imagery conditions. The only difference between the two conditions is the instructional content that accompanies the video (i.e., “Observe the video” or “Imagine yourself performing the action as you observe it”). By randomizing the trials for each condition throughout the experiment, researchers are unable to ensure that the effects of the instructions given for one condition do not influence participants’ behavior on other conditions. Specifically, once participants have been told to imagine themselves performing the action as they observe it, it is difficult to be certain that they are not engaging in the more covert behavior when taking part in subsequent PO trials. The instructional content that accompanies action observation videos has been shown to modulate corticospinal excitability (Roosink and Zijdewind, 2010) and, as such, this may have confounded the results of these three studies (Naish et al., 2014). Presenting the trials as blocks, in a set order so that the combined action observation and imagery trials occur after PO trials can control for this issue.

It is common in TMS action observation and imagery research to record MEPs from a control muscle not involved in the execution of the observed/imagined action. The inclusion of a control muscle provides greater efficacy for facilitation effects being specific to the muscles involved in the execution of the observed/imagined action (e.g., Fadiga et al., 1995, 1999). None of the three combined action observation and imagery experiments published to date that have used TMS have included a control muscle against which to compare facilitation effects for the primary muscle of interest. As such, it is currently unknown whether such a muscle-specific facilitation effect would occur in a combined action observation and imagery condition.

The aims of this study were, therefore, to: (i) determine whether combined action observation and imagery of human movement would facilitate corticospinal excitability to a greater extent than either PO or imagery alone; and (ii) establish whether any corticospinal facilitation effect obtained during combined action observation and imagery of human movement was specific to those muscles involved in the performance of the observed/imagined movement. It was hypothesized that: (i) PO alone, imagery alone and combined action observation and imagery would all produce a corticospinal facilitation effect; (ii) combined action observation and imagery would produce a greater corticospinal facilitation effect than either PO alone or imagery alone; and (iii) such corticospinal facilitation effects would only be present in the muscles involved in the observed and/or imagined action.

## Materials and method

### Participants

Nineteen healthy volunteers (nine females) aged 18–45 years (mean age 26.8 years) participated in the experiment. All participants gave their written informed consent to take part and were naïve to the purpose of the experiment. The TMS Adult Safety Screen (Keel et al., 2001) was used to identify any participants who may have been predisposed to possible adverse effects of the stimulation. No participants were excluded from the study based on their questionnaire responses and no discomfort or adverse effects to the stimulation were reported. All participants were right-handed as assessed by the Edinburgh Handedness Inventory (Oldfield, 1971). The protocol for the experiment was approved by the local university ethics committee and the experiment was conducted in accordance with the Declaration of Helsinki (2013).

### Questionnaire measure

Prior to participating in the experiment, participants completed the Vividness of Movement Imagery Questionnaire—2 (VMIQ-2; Roberts et al., 2008) to provide a marker of their imagery vividness. This 36-item questionnaire requires participants to imagine themselves performing different movements from internal, external, and kinesthetic perspectives. Participants rate the clarity of the images that they generate on a five-point Likert scale, with responses ranging from 1 (*perfectly clear and vivid image*) to 5 (*no image at all*). Lower scores on the VMIQ-2 therefore indicate that participants can generate clear and vivid images. Roberts et al. (2008) reported all three scales to be reliable, observing alpha coefficients of 0.95, 0.95 and 0.93 for the external, internal and kinesthetic scales, respectively.

### Electromyographic recordings

Electromyographic (EMG) recordings were collected from the first dorsal interosseous (FDI) and abductor digiti minimi (ADM) muscles of the right hand using bipolar, single differential, surface EMG electrodes (DE-2.1, Delsys Inc, Boston, MA). The electrodes comprised two 10 mm × 1 mm silver bar strips, spaced 10 mm apart. The EMG was recorded with a sampling rate of 2 kHz, bandwidth 20 Hz to 450 kHz, 92 dB common mode rejection ratio, and >10^15^ Ω input impedance. All electrode sites were cleaned with alcohol swabs prior to electrode attachment. The electrodes were placed over the mid-point of the belly of the muscles and a reference electrode was placed over the ulnar process of the right wrist. The EMG signal was recorded using Spike 2 version 6 software (Cambridge Electronic Design (CED), Cambridge), received by a Micro 1401+ analog-digital converter (CED).

### Transcranial magnetic stimulation

TMS was performed with a figure-of-eight coil (mean diameter of 70 mm) connected to a Magstim 200^2^ magnetic stimulator (Magstim Co., Whitland, Dyfed, UK) which delivered monophasic pulses with a maximum field strength of 2.2 Tesla. The coil was held in a fixed position, using a mechanical arm, over the left motor cortex. The coil was orientated so that the flow of induced current in the brain traveled in a posterior-anterior direction, perpendicular to the central sulcus; the optimal orientation for achieving indirect trans-synaptic activation (Brasil-Neto et al., 1992). The optimal scalp position (OSP) was identified as the scalp site which produced MEPs of the largest amplitude from the right FDI muscle, whilst also eliciting consistent MEPs from the ADM muscle, using a stimulation intensity of 60% maximum stimulator output. The process of stimulating the OSP for the primary muscle of interest and recording MEPs from more than one muscle is common in TMS action observation and imagery research (Naish et al., 2014). The use of 60% maximum stimulator output as the intensity for locating the OSP is also common in research of this nature (e.g., Clark et al., 2004; Loporto et al., 2012; Williams et al., 2012) and is appropriate as it produces large, short-latency MEPs in most individuals. Participants wore a tightly-fitting polyester cap on their head on which the OSP was marked to ensure a constant coil positioning throughout the experiment. The stimulation intensity was then reduced or increased until the resting motor threshold (RMT) was determined. RMT was determined using the MEP amplitudes obtained from the FDI muscle and was defined as the minimum stimulation intensity that elicited peak-to-peak MEP amplitudes greater than 50 μv in at least 5 out of 10 trials (Rossini et al., 1994). As Loporto et al. (2013) demonstrated that facilitation of corticospinal excitability during action observation was only evident following low-intensity TMS, the experiment was conducted at a stimulation intensity of 110% RMT, thereby reducing the chance of direct wave stimulation more frequently seen at higher stimulation intensities.

### Experimental procedures

Participants were seated in a dimly illuminated room in a comfortable chair with their elbows flexed at 90° and their hands placed in a relaxed position on a table in front of them. The participants’ head rested on a chin and head rest to restrict movement. A 37 inch Panasonic LCD television screen (resolution, 1024 × 768 pixels; refresh frequency, 60 Hz) was positioned at a distance of 40 inches from the participant. Participants were requested to refrain from any voluntary movement and to attend to the stimuli presented on the television screen. Blackout curtains ran along either side of the table and behind the screen to eliminate any distractive visual stimuli in the room.

Participants took part in six different conditions (three experimental and three control conditions). The three experimental conditions were termed PO, Movement Imagery (MI), and Combined Action Observation and Movement Imagery (AO+MI). The PO condition showed the dorsal view of a hand in prone position performing six abductions of the index finger at a frequency of 1.33 Hz and participants were instructed to watch the videos. In the MI condition, participants were presented with a blank screen and were instructed to imagine that they were performing index finger abduction movements in time with an auditory metronome at a frequency of 1.33 Hz. In this condition participants were instructed to focus specifically on kinesthetic imagery (i.e., imagining the physiological sensations associated with executing the index finger abduction movement), as this type of imagery has been shown to modulate corticospinal excitability to a greater extent than visual imagery alone (Stinear et al., 2006). In the AO+MI condition, participants observed identical videos to those used in the PO condition, but were instructed to imagine that they were performing the movement as they observed it. As in the MI condition, participants were again instructed to use kinesthetic imagery. In the PO and AO+MI conditions, participants observed the movement being performed by both male and female hands, irrespective of their own sex. The three control conditions were termed Static Hand (SH), Movement Observation (MO), and Backwards Counting (BC). In the SH condition participants were shown the dorsal view of a hand resting in a prone position and instructed to watch the video. In the MO condition participants were instructed to watch a video of pendulum swinging at 1.33 Hz, mimicking the motion of the index finger in the PO and AO+MI conditions. In the BC condition participants observed a blank screen (as in the MI condition), but were instructed to complete a task of counting backwards mentally from a random number, in time with an auditory metronome at 1.33 Hz. All videos were of nine-second duration.

### Experimental protocol

**Figure 1:**
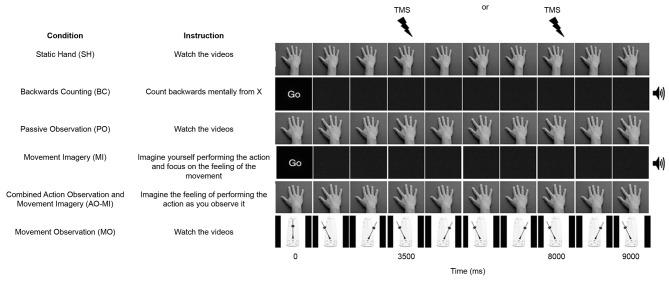
**A schematic representation of the six conditions in the experiment**. All videos were 9000 ms duration and one stimulation was delivered per trial at either 3500 or 8000 ms. An auditory metronome was present during the Backward Counting (BC) and Movement Imagery (MI) conditions.

Participants observed six blocks of trials, with each block containing sixteen videos of the same condition (see Figure 1). The blocks were presented in a semi-random order, where the SH block was always presented before the PO block, the PO block was always presented before the MI block, and the AO+MI block was always presented after both the PO and MI blocks. The purpose of this was to prevent participants from engaging in combined imagery and observation during PO trials or engaging in imagery during SH trials, that could have resulted from having been previously exposed to these experimental conditions. Prior to each block of trials, TMS was delivered during eight pre-block control videos of a blank screen with a fixation cross in order to control for any coil movement between blocks. A single TMS pulse was applied during each video over the OSP at either 3500 or 8000 ms after video onsets. These timings corresponded to the point of maximal abduction in the PO and AO+MI videos. The variation in the onset of the TMS pulse was to remove the predictability of the stimulus. Two-minute rest periods were provided between blocks.

### Data analysis

A pre-stimulus recording of 200 ms was used to check for the presence of EMG activity before the TMS pulse was delivered. Individual trials in which the peak-to-peak amplitude of the baseline EMG activity was 2.5 SD higher than the mean baseline EMG activity of each participant were discarded from further analysis (e.g., Loporto et al., 2012, 2013) since it may have influenced the amplitude of the subsequent MEP. This resulted in 3.4% of trials being discarded from the FDI muscle and 2% of trials being discarded from the ADM muscle.

Due to the nature of the study trials could not be fully randomized across blocks, since the AO+MI videos needed to be presented after the PO videos to prevent participants from engaging in combined imagery and observation during the PO trials. Therefore a 2 (muscle) × 6 (block) repeated measures ANOVA was performed to ensure that there was no change in pre-block (fixation cross) data throughout the experiment to account for any possible coil movement across the conditions that may have affected the MEP results.

The peak-to-peak MEP amplitude was measured from each individual trial and the mean MEP amplitude was calculated for each condition. Due to the large inter-participant variability in absolute MEP amplitudes, these data were normalized using the *z*-score transformation (e.g., Fadiga et al., 1995; Loporto et al., 2012). The normalized MEP amplitudes recorded from both muscles were analyzed using a repeated measures ANOVA, with main factors of muscle (FDI, ADM), and video (SH, PO, MI, AO, BC, MO). *Post hoc* analyses with the Sidak adjustment were applied where necessary. The level of statistical significance for all analyses was set to *α* = 0.05. Effect sizes are reported as partial eta squared ($\eta_{p}^{2}$).

## Results

### VMIQ-2 questionnaire

Participants’ responses to the VMIQ-2 questionnaire revealed mean scores of 28.74 (±13.51) for external visual imagery, 22.26 (±8.22) for internal visual imagery, and 26 (±9.27) for kinesthetic imagery. This indicates that all participants reported being able to generate “reasonably clear and vivid” imagery for all three sub-scales of the questionnaire.

### Pre-block fixation cross data

The results of the 2 (muscle) × 6 (block) repeated measures ANOVA performed on the pre-block (fixation cross) data showed no significant main effects for muscle *F*_(1,18)_ = 1.55, *p* = 0.23, $\eta_{p}^{2}$ = 0.08 or block *F*_(5,90)_ = 0.88, *p* = 0.50, $\eta_{p}^{2}$ = 0.05. In addition, there was no significant muscle × block interaction effect *F*_(5,90)_ = 1.02, *p* = 0.41, $\eta_{p}^{2}$ = 0.05. This confirmed that any MEP amplitude differences found between experimental blocks could be attributed to the video condition presented to the participants, rather than due to any significant coil movement or attentional fatigue across the experiment that may have affected the MEP results.

### Main experiment data

**Figure 2:**
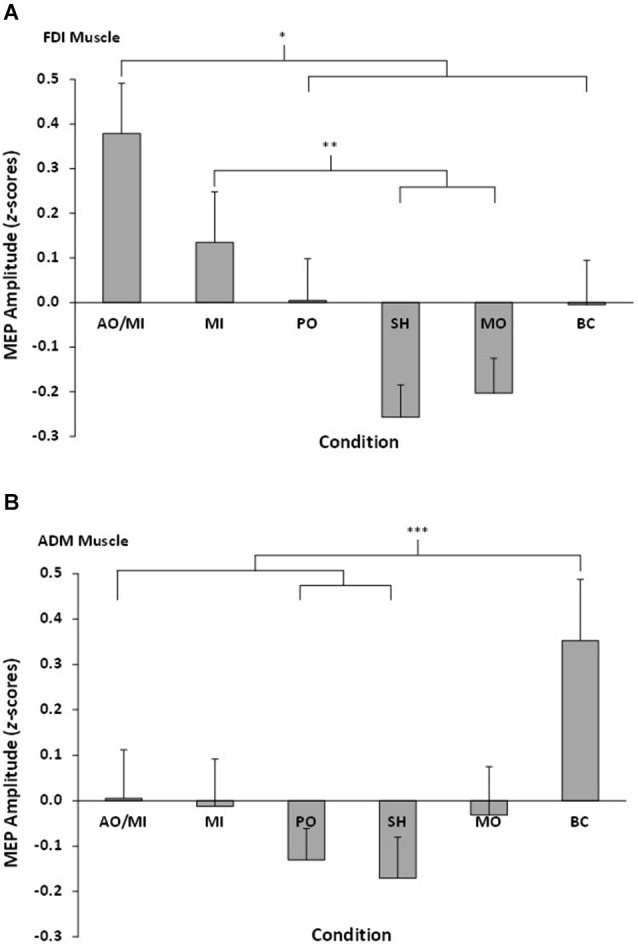
**Mean MEP amplitudes, displayed as** ***z*-scores, recorded from all six conditions for (A) the right FDI muscle and (B) the right ADM muscle**. Asterisks indicate significant differences between conditions.

The repeated measures ANOVA revealed a significant muscle × video interaction effect *F*_(5,90)_ = 4.32, *p* = 0.001, $\eta_{p}^{2}$ = 0.19 (see Figure 2). Pairwise comparisons showed MEP amplitudes recorded from the FDI muscle during AO+MI were significantly higher than PO (*p* = 0.04) and all three control conditions (all *p* < 0.05). There was no significant difference between AO+MI and MI (*p* = 0.15). MEP amplitudes recorded from the FDI muscle during MI were significantly higher than during the control conditions of SH (*p* = 0.01) and MO (*p* = 0.05). There was no significant difference between MI and PO (*p* = 0.45) and MI and BC (*p* = 0.44). In addition, there was no difference between MEP amplitudes obtained during PO in comparison to all three control conditions, although the difference between PO and SH approached significance (*p* = 0.07). No other pairwise comparisons were significant (all *p* > 0.05).

Pairwise comparisons showed MEP amplitudes recorded from the ADM during BC were significantly higher than SH (*p* = 0.01), PO (*p* = 0.007), and AO+MI (*p* = 0.03). No other significant differences were found (all *p* > 0.05).

## Discussion

The primary aim of this experiment was to establish whether combined action observation and imagery of human movement would facilitate corticospinal excitability, and whether such an effect would be greater than that which occurs during either PO or MI alone. The secondary aim was to determine whether any such corticospinal facilitation was specific to the muscles involved in the observed/imagined action. This section will first discuss the current findings in relation to the effects of combined action observation and MI on corticospinal excitability. This will be followed by a discussion of the findings related to PO alone and MI alone, before finally discussing the findings reported in the ADM muscle.

### Facilitation of corticospinal excitability during combined Action Observation and Movement Imagery (AO+MI)

Combined action observation and movement imagery (AO+MI) of simple index finger movements produced larger amplitude MEPs in the FDI muscle than were obtained from control conditions of observing a SH, observing movement of an inanimate object (MO), and counting backwards mentally (BC). The combined action observation and imagery condition also produced MEPs of larger amplitude than passive observation alone (PO). Changes in MEP amplitude represent modulation of corticospinal excitability (Rothwell, 1997; Petersen et al., 2003; Naish et al., 2014). The results therefore indicate that combined action observation and imagery of simple human movements can facilitate corticospinal excitability, and the extent of this facilitation is greater than occurs during PO alone. This finding is consistent with our hypothesis and previous research into the effects of combined action observation and imagery on corticospinal excitability (e.g., Sakamoto et al., 2009; Ohno et al., 2011; Tsukazaki et al., 2012). This facilitation effect during AO+MI was, however, only evident in the FDI muscle, and not the ADM muscle. The FDI muscle is the prime mover in index finger abduction, whilst the ADM is not involved in the execution of this movement. The results, therefore, indicate that the corticospinal facilitation effect during combined AO+MI is specific to the muscles involved in executing the observed/imagined task. Although this effect has been reported in previous action observation (e.g., Fadiga et al., 1995) and imagery (e.g., Fadiga et al., 1999) studies using TMS, to the best our knowledge this study is the first to report such effects in a combined AO+MI condition.

Facilitation of corticospinal excitability during AO+MI may be indicative of activity within the human mirror neuron system. This system, comprising a network of brain regions including the premotor cortex and inferior parietal lobule (Rizzolatti and Craighero, 2004), is activated during both physical movement execution and by observation and imagery of the same action (Rizzolatti, 2005). Although the motor cortex, stimulated in the current experiment, is external to this network of brain regions, Fadiga et al. (2005) proposed that strong cortico-cortical connections link the premotor and motor cortices. It is, therefore, generally accepted that the facilitation of corticospinal excitability during action observation or MI is reflective of increased activity in premotor brain regions that connect to the primary motor cortex (Fadiga et al., 2005). As similar parts of the premotor cortex are activated when observation or imagery are performed in isolation (e.g., Grèzes and Decety, 2001; Filimon et al., 2007; Munzert et al., 2008), engaging concurrent AO+MI may result in stronger activity in these regions (e.g., Macuga and Frey, 2012; Nedelko et al., 2012; Villiger et al., 2013). This may explain the greater facilitation of corticospinal excitability reported for combined AO+MI, compared to PO alone.

Although combined AO+MI facilitated corticospinal excitability to a greater extent than PO, no effect was found in comparison to MI alone. Figure 2 indicates that whilst MEP amplitudes in the combined AO+MI condition appeared to be larger than those obtained in the MI condition, the difference was not significant. This finding conflicts with our hypothesis and previous TMS research which has compared the effects of combined AO+MI against MI alone (e.g., Sakamoto et al., 2009; Tsukazaki et al., 2012). One possible explanation for this inconsistency could be related to discrepancies between the more detailed imagery instructions provided to participants in the current study compared to those offered in previous experiments. Since Stinear et al. (2006) have demonstrated that kinesthetic imagery is more effective in facilitating corticospinal excitability than visual imagery, we instructed participants to focus specifically on “imagining the physiological sensations associated with execution of the index finger abduction movement”. Kinesthetic aspects of imagery, however, were not emphasized in the studies conducted by Sakamoto et al. (2009) and Tsukazaki et al. (2012). For example, Sakamoto et al. told participants to “imagine flexing and extending their elbow”, whilst Tsukazaki et al. told participants to “imagine that they were performing three-ball juggling by mirroring what they saw in the video clips”. It is possible that the instruction to focus on kinesthetic imagery could have enhanced the amplitude of MEPs that we recorded during MI and, as such, contributed to the lack of significant difference in MEP amplitude between combined AO+MI and MI alone. Further controlled work on instructional sets as important mediators of MEP response is clearly warranted.

An alternative explanation for the lack of a significant difference between combined AO+MI and MI alone could be related to the imagery abilities of the participants in the different studies. Williams et al. (2012) correlated MEP amplitudes obtained during imagery of finger-thumb opposition movements with self-reported imagery vividness scores, as measured by the VMIQ-2. They demonstrated that larger amplitude MEPs were associated with a greater kinesthetic imagery vividness. The participants in the current study were all competent imagers, having reported being able to generate “reasonably clear and vivid” images on all sub-scales of the VMIQ-2. Sakamoto et al. (2009) did not report any imagery ability values for participants in their study, whilst the novice jugglers in the study by Tsukazaki et al. (2012) appeared to have a moderate imagery vividness, as measured by a simple self-report measure. It is possible that the participants recruited for this study were more competent imagers than those recruited by Sakamoto et al. and Tsukazaki et al. The possible superior imagery vividness of our participants may have increased MEP amplitudes obtained during MI alone and thus contributed to the lack of difference between combined AO+MI and MI alone conditions. This proposal highlights the importance for researchers to report their participants’ imagery ability characteristics to control for this potentially confounding variable that could inflate MEP contrasts for poor imagers.

### Facilitation of corticospinal excitability during Passive Observation (PO)

It is commonly reported that PO of human movement facilitates corticospinal excitability compared to control conditions (e.g., Fadiga et al., 1995; Strafella and Paus, 2000; Patuzzo et al., 2003; Borroni et al., 2005; Aglioti et al., 2008; Loporto et al., 2012). Despite a trend for this effect (PO > SH; *p* = 0.07), the results of this study do not fully support previous work as PO did not produce MEPs of significantly larger amplitude than the control conditions. This may relate, in part, to the instructions provided to direct participants’ attention to the observation video. The instructions that accompany action observation conditions in TMS research are typically vague and are usually not reported in detail. It is interesting to note, however, that where studies have compared the effects of different instructions during action observation directly, they have often failed to detect a facilitation effect during PO conditions. For example, several researchers have reported that instructing participants to observe an action and simultaneously imagine performing that action facilitates corticospinal excitability, but instructions to only observe an action do not (Sakamoto et al., 2009; Ohno et al., 2011; Tsukazaki et al., 2012). In addition, Roosink and Zijdewind (2010) demonstrated that instructing participants to observe an action with the intention to imitate it later produced MEPs of larger amplitude than when participants were instructed to simply observe an action. These findings are also supported by fMRI research indicating greater activity, compared to PO, in movement-related brain regions when observation and imagery occur simultaneously (e.g., Macuga and Frey, 2012; Nedelko et al., 2012; Villiger et al., 2013) or when actions are observed with the intention of future imitation (e.g., Grèzes et al., 1999; Buccino et al., 2004; Frey and Gerry, 2006). The instructions provided to participants seem to play a crucial role in modulating activity of the motor system during action observation (Naish et al., 2014). Therefore, it is possible that, in some cases, PO alone is not sufficient to enhance corticospinal excitability above resting levels. As such, supplementing PO with additional instructions may be more appropriate in motor rehabilitation settings than only instructing patients to observe a video. Based on the results of this study, and the behavioral evidence provided by Eaves et al. (2014), providing additional instructions for participants to imagine performing the action as they observe it would also appear to be a promising option. Further research should investigate this possibility further by comparing the effects on corticospinal excitability of different types of instructions during observation (e.g., observe and imagine, observe to imitate) against PO.

### Facilitation of corticospinal excitability during Movement Imagery (MI)

Research investigating the effects of MI on corticospinal excitability has shown that imagery of human movement elicits MEPs of larger amplitude than control conditions (e.g., Kasai et al., 1997; Fadiga et al., 1999; Hashimoto and Rothwell, 1999; Rossini et al., 1999; Facchini et al., 2002). The amplitudes of MEPs recorded during imagery, however, do not typically differ from those obtained during PO (e.g., Clark et al., 2004; Léonard and Tremblay, 2007; Roosink and Zijdewind, 2010; Williams et al., 2012). The results of this experiment are consistent with these findings. Despite this, it is important to note that MI did not produce MEPs of larger amplitude than the BC control condition. In previous research, MEP amplitudes obtained during MI have typically been compared to resting MEP values. This comparison, however, does not allow researchers to attribute the facilitation to imagery of human movement *per se*, as the effect may be due to the presence of cognitive activity in the imagery condition. The BC condition was included to address this issue by allowing a comparison to be made between movement-related and non-movement-related cognitive activity. As there was no difference between these two cognitive conditions, it could be argued that the current results do not represent a true corticospinal facilitation effect for MI. Interestingly, Clark et al. (2004) also included a BC condition in their comparison of MEP amplitudes between observation and imagery. Consistent with our findings, they reported that the MEPs obtained during BC were not significantly different to those obtained during imagery or observation. As such, they concluded that part of the facilitation recorded during imagery and observation may be due to attentional processing. The findings reported in both the current study and by Clark et al. indicate that neither PO or MI facilitated corticospinal excitability to a greater extent than a simple non-motor cognitive task. This, therefore, adds weight to the claim that combined AO+MI may be more effective in motor rehabilitation settings than either PO or imagery alone (e.g., Sakamoto et al., 2009; Ohno et al., 2011; Vogt et al., 2013), as combined AO+MI was the only experimental condition to facilitate corticospinal excitability to a greater extent than all three control conditions.

### Facilitation of corticospinal excitability in the ADM muscle

A final point for discussion relates to the findings reported in the ADM muscle. The ADM is not involved in the execution of the experimental task, and so no significant differences between any conditions were expected in this muscle. The amplitude of MEPs recorded during the BC condition were, however, larger than those obtained in SH, PO, and combined AO+MI conditions. This finding can be explained by research indicating a link between counting and hand motor areas. Andres et al. (2007) applied single-pulse TMS to the right hand representation of the motor cortex during counting tasks and a color-recognition control task. They obtained MEPs of larger amplitude during counting conditions, compared to the control task. In a subsequent experiment, they demonstrated that this effect was specific to the hand muscles, as similar findings were not obtained when arm and foot muscles were stimulated during counting. The authors suggested that the explanation for this finding may relate to finger movements playing a crucial role in learning to count during childhood. As a result of this developmental process, hand motor circuits may assist counting in adults by monitoring the relationship between different digits in a series (Andres et al., 2008). The BC condition may therefore have induced, either consciously or sub-consciously, imagined finger movements in the form of “finger counting”. This activity would likely involve the ADM muscle, which may explain why MEP amplitudes were facilitated in this condition. Despite this explanation, it remains unclear why this effect was not evident in the FDI muscle during the BC condition. It is possible, however, that any effects in the FDI were dwarfed by the muscle-specific facilitation effect obtained during observation/imagery of the index finger abduction movement. This link between counting and motor areas may also provide an additional explanation for the lack of difference between MI and BC in FDI muscle, discussed above.

### Limitations

The results of the current experiment provide convincing evidence that combined action observation and MI facilitates corticospinal excitability, but it is important to acknowledge several limitations to the experiment. First, as experimental conditions were presented in a specific order (i.e., SH, then PO, then MI, then AO+MI), participants may have been more familiar with the observed action when they completed the AO+MI condition, compared to when they completed the PO condition. The increased familiarity with the observed movement may, possibly, have contributed to the increased MEP amplitude in the combined condition. However, presenting the conditions in this order was essential in order to discourage participants from engaging in AO+MI during the PO condition.

Second, we cannot confirm that participants did not engage in AO+MI during PO conditions, despite the order of the conditions being structured in an attempt to prevent this. This is a recognized problem in action observation and imagery experiments, as researchers can never be certain that participants complete the conditions exactly as instructed. However, the significant difference between AO+MI and PO conditions indicates that imagery during PO trials is unlikely to have occurred in the current study.

Third, in the MI condition, participants completed their imagery in time with an auditory metronome. The purpose of this was to ensure that the timing of participants imagined finger movements was consistent with the timing of the observed movements in the PO and AO+MI conditions. The auditory metronome was also included in the BC condition as a control. This may be problematic as processing an auditory beat has been shown to activate motor regions in the brain (e.g., Grahn and Brett, 2007). As such, auditory processing, introduced by the presence of the metronome, may have influenced the amplitude of the MEP in the MI and BC conditions. This may account for the lack of significant difference between these conditions. Taken together, however, the inclusion of the metronome was unavoidable given the need to deliver TMS at consistent timings in the imagery and observation conditions.

### Summary

The results presented here have relevance for rehabilitation programs seeking to promote recovery of motor functioning in patients. In stroke rehabilitation settings, PO and MI are both advocated to be beneficial intervention techniques as they can maintain activity in the motor regions of the brain when physical movement is limited or not possible (Sharma et al., 2006; de Vries and Mulder, 2007; Ertelt et al., 2007; Holmes and Ewan, 2007; Mulder, 2007). In the current study, the combined AO+MI condition produced MEPs of larger amplitude than PO, and was the only experimental condition to facilitate corticospinal excitability to a greater extent than all three control conditions. The results therefore indicate that combining observation and imagery techniques into a single intervention strategy may prove to be a more effective tool in rehabilitation settings than use of either technique in isolation.

## Conflict of interest statement

The authors declare that the research was conducted in the absence of any commercial or financial relationships that could be construed as a potential conflict of interest.

## References

[B1] Aglioti S. M. Cesari P. Romani M. Urgesi C. (2008). Action anticipation and motor resonance in elite basketball players. Nat. Neurosci. 11, 1109–1116. 10.1038/nn.2182 19160510

[B2] Andres M. Olivier E. Badets A. (2008). Actions, words and numbers: a contribution to semantic processing?. Curr. Dir. Psychol. Sci. 17, 313–317 10.1111/j.1467-8721.2008.00597.x

[B3] Andres M. Seron X. Olivier E. (2007). Contribution of hand motor circuits to counting. J. Cogn. Neurosci. 19, 563–576. 10.1162/jocn.2007.19.4.563 17381248

[B4] Berends H. I. Wolkorte R. Ijzerman M. J. van Putten M. J. A. M. (2013). Differential cortical activation during observation and observation-and-imagination. Exp. Brain Res. 229, 337–345. 10.1007/s00221-013-3571-8 23771606

[B5] Borroni P. Montagna M. Cerri G. Baldissera F. (2005). Cyclic time course of motor excitability modulation during the observation of a cyclic hand movement. Brain Res. 1065, 115–124. 10.1016/j.brainres.2005.10.034 16297887

[B6] Brasil-Neto J. P. Cohen L. G. Panizza M. Nilsson J. Roth B. J. Hallett M. (1992). Optimal focal transcranial magnetic activation of the human motor cortex: effects of coil orientation, shape of the induced current pulse and stimulus intensity. J. Clin. Neurophysiol. 9, 132–136. 10.1097/00004691-199201000-00014 1552001

[B7] Buccino G. Vogt S. Ritzl A. Fink G. R. Zilles K. Freund H. J.. (2004). Neural circuits underlying imitation learning of hand actions: an event-related fMRI study. Neuron 42, 323–334. 10.1016/S0896-6273(04)00181-3 15091346

[B8] Clark S. Tremblay F. Ste-Marie D. (2004). Differential modulation of corticospinal excitability during observation, mental imagery and imitation of hand actions. Neuropsychologia 42, 105–112. 10.1016/s0028-3932(03)00144-1 14615080

[B54] Declaration of Helsinki. (2013). World Medical Association Declaration of Helsinki: ethical principles for medical research involving human subjects. JAMA 310, 2191–2194. 10.1001/jama.2013.281053 24141714

[B9] de Vries S. Mulder T. (2007). Motor imagery and stroke rehabilitation: a critical discussion. J. Rehabil. Med. 39, 5–13. 10.2340/16501977-0020 17225031

[B10] Eaves D. L. Haythornthwaite L. Vogt S. (2014). Motor imagery during action observation modulates automatic imitation effects in rhythmical actions. Front. Hum. Neurosci. 8:28. 10.3389/fnhum.2014.00028 24600369 PMC3927126

[B11] Ertelt D. Small S. Solodkin A. Dettmers C. McNamara A. Binkofski F.. (2007). Action observation has a positive impact on rehabilitation of motor deficits after stroke. Neuroimage 36, T164–T173. 10.1016/j.neuroimage.2007.03.043 17499164

[B12] Facchini S. Muellbacher W. Battaglia F. Boroojerdi B. Hallett M. (2002). Focal enhancement of motor cortex excitability during motor imagery: a transcranial magnetic stimulation study. Acta Neurol. Scand. 105, 146–151. 10.1034/j.1600-0404.2002.1o004.x 11886355

[B13] Fadiga L. Buccino G. Craighero L. Fogassi L. Gallese V. Pavesi G. (1999). Corticospinal excitability is specifically modulated by motor imagery: a magnetic stimulation study. Neuropsychologia 37, 147–158. 10.1016/S0028-3932(98)00089-X 10080372

[B14] Fadiga L. Craighero L. Olivier E. (2005). Human motor cortex excitability during the perception of others’ action. Curr. Opin. Neurobiol. 15, 213–218. 10.1016/j.conb.2005.03.013 15831405

[B15] Fadiga L. Fogassi L. Pavesi G. Rizzolatti G. (1995). Motor facilitation during action observation: a magnetic stimulation study. J. Neurophysiol. 73, 2608–2611. 7666169 10.1152/jn.1995.73.6.2608

[B16] Filimon F. Nelson J. D. Hagler D. J. Sereno M. I. (2007). Human cortical representations for reaching: mirror neurons for execution, observation and imagery. Neuroimage 37, 1315–1328. 10.1016/j.neuroimage.2007.06.008 17689268 PMC2045689

[B17] Frey S. H. Gerry V. E. (2006). Modulation neural activity during observational learning of actions and their sequential orders. J. Neurosci. 26, 13194–13201. 10.1523/JNEUROSCI.3914-06.2006 17182769 PMC6674989

[B18] Grahn J. A. Brett M. (2007). Rhythm and beat perception in the motor areas of the brain. J. Cogn. Neurosci. 19, 893–906. 10.1162/jocn.2007.19.5.893 17488212

[B19] Grèzes J. Costes N. Decety J. (1999). The effects of learning and intention on the neural network involved in the perception of meaningless actions. Brain 122, 1875–1887. 10.1093/brain/122.10.1875 10506090

[B20] Grèzes J. Decety J. (2001). Functional anatomy of execution, mental simulation, observation and verb generation of actions: a meta-analysis. Hum. Brain Mapp. 12, 1–19. 10.1002/1097-0193(200101)12:1<01::AID-HBM10>3.0.CO;2-V 11198101 PMC6872039

[B21] Hashimoto R. Rothwell J. C. (1999). Dynamic changes in corticospinal excitability during motor imagery. Exp. Brain Res. 125, 75–81. 10.1007/s002210050660 10100979

[B22] Holmes P. Calmels C. (2008). A neuroscientific review of imagery and observation use in sport. J. Mot. Behav. 40, 433–445. 10.3200/JMBR.40.5.433-445 18782718

[B23] Holmes P. Ewan L. (2007). The use of structured observation as a stroke rehabilitation aid: an opinion from neuroscience. Brit. J. Occupat. Ther. 70, 454–456.

[B24] Kasai T. Kawai S. Kawanishi M. Yahagi S. (1997). Evidence for facilitation of motor evoked potentials (MEPs) induced by motor imagery. Brain Res. 744, 147–150. 10.1016/S0006-8993(96)01101-8 9030424

[B25] Keel J. C. Smith M. J. Wassermann E. M. (2001). A safety screening questionnaire for transcranial magnetic stimulation. Clin. Neurophysiol. 112:720. 10.1016/S1388-2457(00)00518-6 11332408

[B26] Léonard G. Tremblay F. (2007). Corticomotor facilitation associated with observation, imagery and imitation of hand actions: a comparative study in young and old adults. Exp. Brain Res. 177, 167–175. 10.1007/s00221-006-0657-6 16947064

[B27] Loporto M. Holmes P. S. Wright D. J. McAllister C. J. (2013). Reflecting on mirror mechanisms: motor resonance effects during action observation only present with low-intensity transcranial magnetic stimulation. PLoS One 8:e64911. 10.1371/journal.pone.0064911 23724104 PMC3665751

[B28] Loporto M. McAllister C. J. Edwards M. G. Wright D. J. Holmes P. S. (2012). Prior action execution has no effect on corticospinal facilitation during action observation. Behav. Brain Res. 231, 124–129. 10.1016/j.bbr.2012.03.009 22449863

[B29] Loporto M. McAllister C. Williams J. Hardwick R. Holmes P. (2011). Investigating central mechanisms underlying the effects of action observation and imagery through transcranial magnetic stimuluation. J. Motor. Behav. 43, 361–373. 10.1080/00222895.2011.604655 21861627

[B30] Macuga K. L. Frey S. H. (2012). Neural representations involved in observed, imagined and imitated actions are dissociable and hierarchically organized. Neuroimage 59, 2798–2807. 10.1016/j.neuroimage.2011.09.083 22005592 PMC3254825

[B31] Mulder T. (2007). Motor imagery and action observation: cognitive tools for rehabilitation. J. Neural Transm. 114, 1265–1278. 10.1007/s00702-007-0763-z 17579805 PMC2797860

[B32] Munzert J. Zentgraf K. Stark R. Vaitl D. (2008). Neural activation in cognitive motor processes: comparing motor imagery and observation of gymnastic movements. Exp. Brain Res. 188, 437–444. 10.1007/s00221-008-1376-y 18425505

[B33] Naish K. R. Houston-Price C. Bremner A. J. Holmes N. P. (2014). Effects of action observation on corticospinal excitability: muscle specificity, direction and timing of the mirror response. Neuropsychologia [Epub ahead of print]. 64, 331–348. 10.1016/j.neuropsychologia.2014.09.034 25281883

[B34] Nedelko V. Hassa T. Hamzei F. Schoenfeld M. A. Dettmers C. (2012). Action imagery combined with action observation activates more corticomotor regions than action observation alone. J. Neurol. Phys. Ther. 36, 182–188. 10.1097/NPT.0b013e318272cad1 23095902

[B35] Ohno K. Higashi T. Sugawara K. Ogahara K. Funase K. Kasai T. (2011). Excitability changes in the human primary motor cortex during observation with motor imagery of chopstick use. J. Physical Ther. Sci. 23, 703–706 10.1589/jpts.23.703

[B36] Oldfield R. C. (1971). The assessment and analysis of handedness: the Edinburgh inventory. Neuropsychologia 9, 97–113. 10.1016/0028-3932(71)90067-4 5146491

[B37] Patuzzo S. Fiaschi A. Manganotti P. (2003). Modulation of motor cortex excitability in the left hemisphere during action observation: a single- and paired-pulse transcranial magnetic stimulation study of self- and non-self-action observation. Neuropsychologia 41, 1272–1278. 10.1016/S0028-3932(02)00293-2 12753966

[B38] Petersen N. T. Pyndt H. S. Nielsen J. B. (2003). Investigating human motor control by transcranial magnetic stimulation. Exp. Brain Res. 152, 1–16. 10.1007/s00221-003-1537-y 12879177

[B39] Rizzolatti G. (2005). The mirron neuron system and its function in humans. Anat. Embryol. (Berl) 210, 419–421. 10.1007/s00429-005-0039-z 16222545

[B40] Rizzolatti G. Craighero L. (2004). The mirror-neuron system. Annu. Rev. Neurosci. 27, 169–192. 10.1146/annurev.neuro.27.070203.144230 15217330

[B41] Roberts R. Callow N. Hardy L. Markland D. Bringer J. (2008). Movement imagery ability: development and assessment of a revised version of the vividness of movement imagery questionnaire. J. Sport Exerc. Psychol. 30, 200–221. 18490791 10.1123/jsep.30.2.200

[B42] Roosink M. Zijdewind I. (2010). Corticospinal excitability during observation and imagery of simple and complex hand tasks: implications for motor rehabilitation. Behav. Brain Res. 213, 35–41. 10.1016/j.bbr.2010.04.027 20433871

[B43] Rossini P. M. Barker A. T. Berardelli A. Caramia M. D. Caruso G. Cracco R. Q.. (1994). Non-invasive electrical and magnetic stimulation of the brain, spinal cord and roots: basic principles and procedures for routine clinical application. Report of an IFCN committee. Electroencephalogr. Clin. Neurophysiol. 91, 79–92. 10.1016/0013-4694(94)90029-9 7519144

[B44] Rossini P. M. Rossi S. Pasqualetti P. Tecchio F. (1999). Corticospinal excitability modulation to hand muscles during movement imagery. Cereb. Cortex 9, 161–167. 10.1093/cercor/9.2.161 10220228

[B45] Rothwell J. C. (1997). Techniques and mechanisms of action of transcranial stimulation of the human motor cortex. J. Neurosci. Methods 74, 113–122. 10.1016/S0165-0270(97)02242-5 9219881

[B46] Sakamoto M. Muraoka T. Mizuguchi N. Kanosue K. (2009). Combining observation and imagery of an action enhances human corticospinal excitability. Neurosci. Res. 65, 23–27. 10.1016/j.neures.2009.05.003 19463869

[B47] Sharma N. Pomeroy V. M. Baron J. C. (2006). Motor imagery: a backdoor to the motor after stroke? Stroke 37, 1941–1952. 10.1161/01.STR.0000226902.43357.fc 16741183

[B48] Stinear C. M. Byblow W. D. Steyvers M. Levin O. Swinnen S. P. (2006). Kinesthetic, but not visual, motor imagery modulates corticomotor excitability. Exp. Brain Res. 168, 157–164. 10.1007/s00221-005-0078-y 16078024

[B49] Strafella A. P. Paus T. (2000). Modulation of cortical excitability during action observation: a transcranial magnetic stimulation study. Neuroreport 11, 2289–2292. 10.1097/00001756-200007140-00044 10923687

[B50] Tsukazaki I. Uehara K. Morishita T. Ninomiya M. Funase K. (2012). Effect of observation combined with motor imagery of a skilled hand-motor task on motor cortical excitability: difference between novice and expert. Neurosci. Lett. 518, 96–100. 10.1016/j.neulet.2012.04.061 22580208

[B51] Villiger M. Estévez N. Hepp-Raymond M. C. Kiper D. Kollias S. S. Eng K.. (2013). Enhanced activation of motor execution networks using action observation combined with imagination of lower limb movements. PLoS One 8:e72403. 10.1371/journal.pone.0072403 24015241 PMC3756065

[B52] Vogt S. Di Rienzo F. Collet C. Collins A. Guillot A. (2013). Multiple roles of motor imagery during action observation. Front. Hum. Neurosci. 7:807. 10.3389/fnhum.2013.00807 24324428 PMC3839009

[B53] Williams J. Pearce A. J. Loporto M. Morris T. Holmes P. S. (2012). The relationship between corticospinal excitability during motor imagery and motor imagery ability. Behav. Brain Res. 226, 369–375. 10.1016/j.bbr.2011.09.014 21939692

